# The impact of fangxian huangjiu on the fermentation quality and microbial community dynamics of cigar wrapper leaves

**DOI:** 10.3389/fbioe.2024.1428750

**Published:** 2024-07-25

**Authors:** Lan Yao, Tongtong Zhang, Yule Shan, Jingpeng Yang, Jun Yu, Chunlei Yang, Xiong Chen

**Affiliations:** ^1^ Key Laboratory of Fermentation Engineering (Ministry of Education), Cooperative Innovation Center of Industrial Fermentation (Ministry of Education and Hubei Province), College of Life and Health Science, Hubei University of Technology, Wuhan, China; ^2^ Tobacco Research Institute of Hubei Province, Wuhan, China

**Keywords:** cigar wrapper leaves, fangxian huangjiu, volatile aroma components, metagenomics, microbial community dynamics

## Abstract

**Introduction:** Cigar wrapper leaves (CWLs) plays a crucial role in reflecting cigar overall quality. Originating from the Qinba region of China, Fangxian Huangjiu (FHJ) is distinctive from other varieties of Huangjiu.

**Methods:** To investigate the effects of FHJ on enhancing the aroma and quality of CWLs, as well as the consequent alterations in microbial communities, Gas Chromatography-Mass Spectrometry (GC-MS) coupled with Odor Active Value (OAV) analysis was utilized to evaluate the volatile aroma components of CWLs.

**Results and Discussion:** The results indicated that the total amount of aroma compounds in CWLs reached 3,086.88 ug/g, increasing of 270.50% and 166.31% compared to the unfermented and naturally fermented groups, respectively. Among them, β-ionone and 4,7,9-megastigmatrien-3-one from the FHJ fermentation group significantly influenced the sensory characteristics of CWLs. Metagenomic results demonstrated that FHJ fermentation enriched the abundance of both shared and unique microbial species in CWLs, while also increased the diversity of differential microbial species. Addition of FHJ effectively altered the microbial community structure of CWLs from a dominance of *Staphylococcus* to a prevalence of *Staphylococcus*, *Aspergillus*, *Pseudomonas*, and *Acinetobacter*. The interactions among these diverse microorganisms collectively contribute to the enhancement of the intrinsic quality of CWLs. This paper provides a theoretical basis for improving the quality of CWLs by FHJ and exploring the changes of microbial community structure and interaction between CWLs and FHJ.

## 1 Foreword

Cigars are a kind of tobacco product, characterized by a rich aroma distinct from cigarettes, with flavor profiles ranging from savory to sweet, and typically delivering a strong nicotine hit (Hu et al., 2022a). CWLs serve as an external symbol of the cigar’s quality (Zhang et al., 2024). During the smoking process, the CWLs come into direct contact with air, facilitating a more intense combustion and thus contributing significantly to the overall sensory experience. Consequently, the industry demands high standards for both the appearance and intrinsic quality of CWLs. Currently, fermentation is recognized as a crucial process for enhancing both the external appearance and internal quality of CWLs. Enzymatic reactions occurring during fermentation facilitate the development of color and formation of intrinsic aroma compounds in CWLs, along with alterations in their conventional chemical composition. CWLs tend to be alkaline, and the pH level significantly influences the nicotine content. Higher pH levels result in elevated nicotine content. Excessive nicotine not only adversely affects the smoking experience but also poses health risks (Torikai et al., 2004).

Huangjiu, also known as Chinese rice wine, is one of the three ancient alcoholic beverages alongside beer and wine (Jin et al., 2021). Traditional Huangjiu is produced through the fermentation of various grains such as rice, sorghum, glutinous rice, corn, millet, and barley, under the action of koji. It undergoes a four-stage fermentation process, resulting in a mild taste and elegant aroma. Huangjiu is rich in nutritional value, containing abundant proteins that are enzymatically broken down into amino acids and peptides (Wang et al., 2024). The flavor of Huangjiu is determined by both taste and aroma, with taste coming from sugars, organic acids, amino acids and other substances, in which the volatile flavor substances in Huangjiu are mainly influenced by alcohols, esters, acids, aldehydes and other components (Li, 2023). The volatile aroma substances in Huangjiu may increase the content of corresponding volatile aroma components in cigar tobacco leaves. When Huangjiu is added to the fermentation of cigar tobacco leaves (CTLs), the volatile flavor substances it contains may have a promoting effect on the aroma enhancement of CTLs. Recently, it was reported that adding “Humi” water of three different roasting degrees to cigar tobacco leaves and co-fermenting them resulted in a significant improvement in the sensory quality of the fermented cigar tobacco leaves compared to natural fermentation. This improvement may be attributed to the substances in the cooked rice water participating in the growth and metabolism of microorganisms, increasing the volatile aroma compounds in the cigar tobacco leaves (Ren et al., 2024).

During the fermentation process of CTLs, surface microorganisms utilize nutrients from the cigar themselves or external sources to decompose large molecular substances such as nicotine, starch, cellulose into smaller molecular substances, which serve as both substrates for microbial survival and precursors for the production of volatile aroma compounds. FHJ, originating from the Qinba region of China, exhibits distinct differences in taste and trace substances compared to traditional Shaoxing Huangjiu. FHJ is characterized by its acidity and rich content of organic acids such as malic acid and citric acid, endowing it with unique flavor and nutritional value (Gao et al., 2024). Hu et al., 2023 (Hu et al., 2022b) added plant extracts mainly composed of loquat to the fermentation of CTLs as a fermentation substrate, with a control group treated only with deionized water. The results showed that the addition of the medium influenced the trend of non-volatile organic acids in CTLs. Furthermore, it facilitated the accumulation of aroma compounds and significantly altered the microbial community structure of CTLs, promoted the formation of aroma compounds. The impact of fermentation media on cigar filler has been studied, revealing significant changes in bacterial diversity and an increase in OTU levels in t CTLs following the addition of culture medium (Ma et al., 2023). In the field of cigar fermentation, the use of exogenous additives in fermenting CTLs remains relatively unexplored, with Huangjiu as an exogenous additive being particularly understudied. The addition of FHJ can effectively lower the pH of CTLs, significantly reducing nicotine content. Moreover, the addition of FHJ might induce changes in the microbial community structure of CTLs, thereby improving their quality. This research aims to investigate the mechanism by which FHJ, as an exogenous additive, affects the physicochemical properties and microbial community structure of CWLs.

## 2 Materials and methods

### 2.1 Experimental materials

The test CWLs samples (CX-026) were harvested in 2022 from the CWLs Station of Enshi Prefecture, Hubei Province, China. The rice wine was provided by Fangzhouhuang RiceWine Factory in Fangzhou, Shiyan City, Hubei Province.

Buffer solution: 100 mmol/L Tris-HCl, 50 mmol/L ethylenediaminetetraacetic acid disodium salt (EDTA-Na2), 20 g/L polyvinylpyrrolidone (PVP), 1 mL/L Tween-20, 1.4 mol/L NaCl, pH = 8.

### 2.2 Experimental methods

#### 2.2.1 Fermentation method

Box fermentation method was employed, with 1.5 m in length, 1.2 m in width, and 1.3 m in height. The weight of CWLs in each box was 100 kg. Industrial fermentation was conducted in a fermentation room maintained at temperature of 30°C–35°C. The initial fermentation temperature of the CWLs was set at 30°C, with a turning pile temperature of 50°C ± 1°C based on our previous studies (Yao et al., 2022a). The blank control group (bf) consisted of CWLs before fermentation, while the control group (ck) underwent natural fermentation with water addition only. The experimental group (z65) involved spraying CWLs with a concentration of 65% FHJ. The initial moisture content of all treatment groups was set at 30% ± 1%. Turning of the CWLs followed the principle of turning from top to bottom and from outer layer to inner layer. The fermentation process lasted for 40 days, during which turning and sampling were conducted three times.

#### 2.2.2 Determination of volatile aroma substances in FHJ and CWLs

6 mL of FHJ was diluted to 14% vol in a 30 mL headspace bottle, which was kept on a 55°C constant-temperature magnetic stirrer to equilibrate for 15 min at a speed of 500 r/min. Injection handle was inserted with the extraction fibers into the headspace bottle. After the extraction was completed, the fiber was inserted into the GC-MS injection port at 260 ted, the fiber was inserted non-split mode. Conditions for the determination of volatile aroma substances of FHJ: Agilent Techologies 7890B gas chromatograph (Agilent Technologies, Santa Clara, CA) and 5977B mass spectrometry detector (7890B–5975B, Agilent Technologies, Inc. Santa Clara, CA), CP-WAX57CB column (50 m × 0.25 mm× 0.20 μm), 50/30 mm DVB/CAR/PDMS extraction fiber (Shanghai Amperexperiment Science and Technology Co., Ltd.), MS-H-ProA LCD digital display heated disc magnetic stirrer (Dalong Xingchuang Experimental Instrument (Beijing) Co.) Ltd.), 30 mL headspace vial (Shanghai Rebus Network Technology Co., Ltd.). The carrier gas was high-purity helium (He), with a constant flow rate of 0.7 mL/min in non-split mode. The initial temperature of the column was held at 45°C for 1.5 min, and then increased to 85°C at a rate of 6°C/min, and then increased to 225°Cat a rate of 4°C/min and kept for 15 min. The temperature of the quadrupole was 150°C, the temperature of the transmission line was 250°C, and the temperature of the ion source was 230°C. The electron energy was 70 eV, full scan mode, and the scanning mass range was 30–550 aum.

10 g of dried CWLs sample powder was processed by simultaneous distillation extraction (SDE) technique, using saturated NaCl solution and dichloromethane as the extraction solvents. After extraction, the extract was collected and concentrated to 2 mL, and 50 μL of phenylethyl acetate (1.2028 mg/mL) was added as the internal standard and analyzed by gas chromatography-mass spectrometry (GC-MS). Conditions for the determination of volatile aroma substances in CWLs: Agilent Techologies 7890A gas chromatograph (Agilent Technologies, Santa Clara, CA) with 5975C mass selective detector (7890A-5975C, Agilent Technologies. Santa Clara, CA), HP-5MS capillary column (30 m × 0.25 mm, 0.25 μm). Injection volume was 1 μL. Carrier gas was helium at a flow rate of 1 mL/min with a shunt ratio of 10:1. The initial oven temperature was set to 40°C, held for 2 min, ramped up to 200°C at a rate of 2°C/min, and held for 5 min, then ramped up at 10°C/min to 280°C. The electron collision energy was 70 eV. The transmission line temperature was 250°C and the ion source temperature was 230°C. The solvent delay time was 3 min.

#### 2.2.3 Calculation of odor active value

The contribution of each volatile compound to the characteristic aroma of CWLs was assessed qualitatively and quantitatively through their OAVs. The OAV was calculated using the equation OAV = C/T, where C represents the total concentration of each volatile aroma compound in CWLs (in µg/g), and T represents the odor threshold of the compound in either water or ethanol solution (in µg/mL), from published literature (Young et al., 1996). The OAV represents the ratio of the concentration of aroma compounds to their odor thresholds. When the OAV value is ≥ 1, the flavor substance is considered as a key flavor compound contributing significantly to the sample’s flavor. The higher of OAV value, the greater of flavor contribution. When 0.1≤ OAV <1, the flavor substance is considered to have some contribution to the sample’s flavor.

#### 2.2.4 Sensory evaluation of cigar tobacco leaves

The fermented CWLs were hand-rolled into cigars for sensory evaluation. The main quality indicators were evaluated by a panel of experts from Tobacco Research Institute of Hubei Province. And the School of Life Sciences and Health Engineering, Hubei University of Technology, using a nine-point system, including seven indicators: aroma quantity, aroma quality, permeability, miscellaneous odor, strength, stimulation and aftertaste.

#### 2.2.5 Collection of microorganisms from the surface of CWLs

30 g of CWLs samples were cut into pieces to ensure sufficient contact with the buffer solution. They were then placed in 300 mL of buffer solution and subjected to 30 min of ultrasound treatment. After discarding the CWLs fragments, the collected liquid was centrifuged at 4°C and 6,000 rpm for 10 min. After resuspending the precipitate, centrifugation was continued, and the supernatant was discarded. This washing process was repeated until the supernatant became nearly colorless. Microbial cells were collected when the precipitate mass exceeded 200 mg. The microbial precipitate was flash-frozen in liquid nitrogen for at least 30 min and then transferred to −80°C for storage.

#### 2.2.6 DNA extraction, library construction, and metagenomic sequencing

Using the extracted DNA as a template, bacterial genes were amplified using the forward primer 515F (5′-GTGCCAGCMGCCGCGGTAA-3′) and the reverse primer 907R (5′-CCGTCAATTCMTTTRAGTTT-3′). Library construction and sequencing were conducted at Beijing Novogene Co., Ltd., with each sample undergoing three replicates.

#### 2.2.7 Data processing

The chromatographic profiles were analyzed using GC-MS solutionver software (Agilent Technologies, United States). Mass spectral data were compared with spectra from the NIST reference library (NIST 14) within the GC-MS data system to identify volatile compounds. The differences in microbial taxa before and after fermentation were processed using the bioinformatics analysis cloud platform of Beijing Novogene Co., Ltd. (https://magic.novogene.com/). Significance analysis was conducted using Statistica 23.0 software (SPSS Inc., Chicago, IL, United States), and multivariate analysis was performed using SIMCA-P14 software (Umetrics, Umeå, Sweden).

## 3 Results

### 3.1 Determination of volatile aromatic compounds in FHJ

FHJ originates from the Qinba region of China, where its unique water source and distinctive natural climate cultivate a unique microbial community for brewing FHJ, making it different from traditional Shaoxing rice wine and Hakka rice wine. Qualitative detection of volatile aroma compounds in rice wine was conducted using HS-SPME-GC-MS technology, revealing a total of 30 aroma compounds (Table 1). Among them, compounds such as benzaldehyde, benzyl alcohol, and furfural contribute sweetness to FHJ, reducing its astringency. The unique aroma of FHJ, characterized by its cleanliness and sweetness, is contributed by compounds like benzyl alcohol. Wang et al. (Wang et al., 2014) utilized HS-SPME-GC-MS analysis to determine the aroma compounds in rice wine during the frying process, revealing that esters, alcohols, and ethers are the most abundant aroma components and major contributors to the flavor of rice wine. The koji used in the fermentation of FHJ is primarily made from the herbaceous plant *Polygonum hydropiper*, resulting in a full-bodied aroma and a sweet taste. In contrast, traditional Shaoxing rice wine is mostly fermented using rice koji and wheat koji, imparting a rich rice aroma and a mild and refreshing taste.

**TABLE 1:** Volatile aroma compounds in Fangxian Huangjiu.

Cas	Aroma compounds	Cas	Aroma compounds
123-51-3	3-Methyl-1-butanol	556-68-3	hexadecamethylcyclooctasiloxane
1000-05-1	1,1,3,3,5,5,7,7-octamethyltetrasiloxane	107-50-6	Tetradecamethyl Cycloheptasiloxane
5617-32-3	heptaethylene glycol	64-17-5	Ethanol
541-05-9	hexamethylcyclotrisiloxane	5779-94-2	2,5-Dimethylbenzaldehyde
1873-88-7	1,1,1,3,5,5,5-Heptamethyltrisiloxane	4747-07-3	N-hexyl methyl ether
1066-42-8	dimethylsilanediol	10348-47-7	ethyl 2-hydroxy-4-methyl valerate
513-85-9	2,3-Butanediol	540-97-6	Dodecamethylcyclohexasiloxane
19095-23-9	1,13-Dihydrotetradecamethylheptasiloxan	79-31-2	Isobutyric acid
1572-93-6	(R)-(-)-3-METHYL-2-BUTANOL	78-83-1	isobutanol
6797-44-0	1-phenyl-1,2,3-butanetrione 2-oxime	64-19-7	acetic acid
1679-47-6	Alpha-Methyl-Gamma-Butyrolactone	123-25-1	Diethyl succinate
19095-24-0	1h,15h-hexadecamethyloctasiloxane	97-64-3	Ethyl lactate
107-92-6	Butyric Acid	100-52-7	Benzaldehyde
111-35-3	3-Ethoxy-1-propanol	98-01-1	Furfural
628-97-7	Ethyl palmitate	60-12-8	Phenethyl alcohol

### 3.2 Effects of different treatment methods on volatile aromatic compounds in CWLs

After fermentation through different treatment, the types and levels of volatile aroma compounds in CWLs undergo varying degree of changes (Table 2). Specifically, the total content of aroma compounds in the bf treatment group was 1,141.19 μg/g. After three rounds of pile turning fermentation, the aroma compound content in the ck-3 group increased to 1856.14 μg/g, representing a cumulative increase of 714.95 μg/g. In the z65 group, following three rounds of pile turning fermentation, the aroma compound content reached 3,086.88 μg/g, which is an increase of 1,945.69 μg/g and 1,230.74 μg/g compared to the bf group and the ck group, respectively. Additionally, the content of carotenoid degradation products increased to 275.81 μg/g after rice wine fermentation treatment, representing an increase of 82.50% and 63.30% compared to the bf group and ck group, respectively.

**TABLE 2:** Content of volatile aroma compounds (µg/g) at four different fermentation stages.

	Aroma compounds	Odor descriptor	Concentration^b^ (µg/g)						
			bf	Ck-1	Ck-2	Ck-3	z65-1	z65-2	z65-3
carotenoid degradation products	4,7,9-Megastigmatrien-3-one	dry fruit	18.97b	71.76a	87.86a	59.02a	72.32a	96.87a	162.35a
	Dihydroactinidiolide	musk	0.7b	6.99b	—	—	5.8a	11.21a	16.68a
	Geranylacetone	magnolia	1.41b	1.73ab	3.37ab	—	2.98a	6.87a	4.56a
	β-Cyclocitral	aroma	0.4	0.57	1.83	0.22	0.94	—	1.77
	β-Damascenone	flowery	5.85	—	20.14	—	10.18	9.59	—
	6-methylhept	mandarin orange	0.42	0.19	—-	0.4	—	0.5	0.32
	β-Ionone	violet	10.42	—	16.9	4.59	7.8	5.83	10.07
	Dihydro-beta-ionone	magnolia	—	—	—	30.08	11.73	—	26.83
	4-(2,6,6-Trimethyl-1-cyclohexenyl)-3-buten-2-ol	flowery	—	8.07	—	6.6	0.69	0.57	2.65
	Geranylgeraniol	rose	9.85	5.48	21.19	0.32	7.37	4.5	50.58
	2,3-Dihydro-2,2,6-trimethylbenzaldehyde	saffron	0.25	0.35	0.34	—	0.38	0.54	—
	total	—	48.27b	95.14ab	151.63ab	101.23ab	120.19a	136.48a	275.81a
Phenylalanine degradation products	Phenethyl alcohol	rose	5.78c	6.68b	19.53b	16.26b	34.72a	45.75a	48.81a
	Benzyl alcohol	flowery	—	—	3.16	4.26	—	—	5.07
	Benzaldehyde	almond	2.55b	2.98ab	3.37ab	6.17ab	2.44a	1.75a	1a
	Phenylacetaldehyde	black tea	14.21b	49.53a	64.64a	38.94a	48.31ab	51.01ab	—
	total	—	22.54 b	59.19a	90.70a	65.63a	85.47a	98.51a	54.88a
maillard reaction products	Furfuryl alcohol	acrimony	—	4.68	—	6.23	11.27	17.66	17.13
	Furfural	almond	—	1.14b	4.36b	2.57b	6.08a	11.21a	7.64a
	5-Methyl furfural	caramel	—	3.24	4.35	7.43	5.5	8.15	2.62
	Indole	flowery	7.89	16.99	15.29	—	—	19.7	17.05
	total	—	7.89b	26.05ab	24.00ab	16.23ab	22.85a	56.72a	44.44a
Cembranoid degradation products	D-Solanone	carrot	19.16b	57.33ab	—	—	56.61a	75.36a	70.43a
	total	—	19.16b	57.33ab	0ab	0ab	56.61a	75.36a	70.43a
chlorophyll degradation products	Neophytadiene		934.32b	1,402.31a	1,163.03a	1,006.92a	1,325.1a	1,436.08a	1883.7a
	total	—	934.32b	1,402.31a	1,163.03a	1,006.92a	1,325.1a	1,436.08a	1883.7a
other	nerolidol acetate	apple	5.14	8.28	2.79	—	13.86	11.92	—
	3,7,11-Trimethyl-1,6,10-dodecatrien-3-ol	flores aurantii	0.7	—	0.98	—	0.13	—	—
	(−)-Isopulegol	peppermint	—	0.5	0.19	—	0.39	0.33	—
	Geranyl linalool	peppermint	5.97c	44.3b	43.84b	104.93b	224.06a	232.74a	173.05a
	Sclareol	ambergris	64.43b	132.3ab	33ab	213.56ab	254.73a	396.07a	202.84a
	Isopulegol	peppermint	—	—	1.56	0.22	0.17	0.50	1.89
	Phytol	flowery	32.76b	306.11a	317.84a	337.55a	288.96a	401.67a	372.86a
	Perilla alcohol	peppermint	—	0.44	—	0.09	0.69	—	0.72
	3-Tolylaldehyde	nut	—	—	—	9.78	16.25	11.38	—
	P-vinylguaiacol	clove	—	2.46b	4.49b	—	4.59a	5.69a	6.26a
	total	—	109c	494.39b	404.69b	666.13b	803.83a	1,060.3a	757.62a
Total	Total amount of aroma substances		1,141.18b	2,134.41a	1834.05a	1856.14a	2,414.05a	2,863.45a	3,086.88a

Value with different letters (a-c) in a column are significantly different using Duncan’s multiple comparison tests (*p* < 0.05).

### 3.3 Calculation of odor active value and its impact on fermentation of CWLs

**FIGURE 1:**
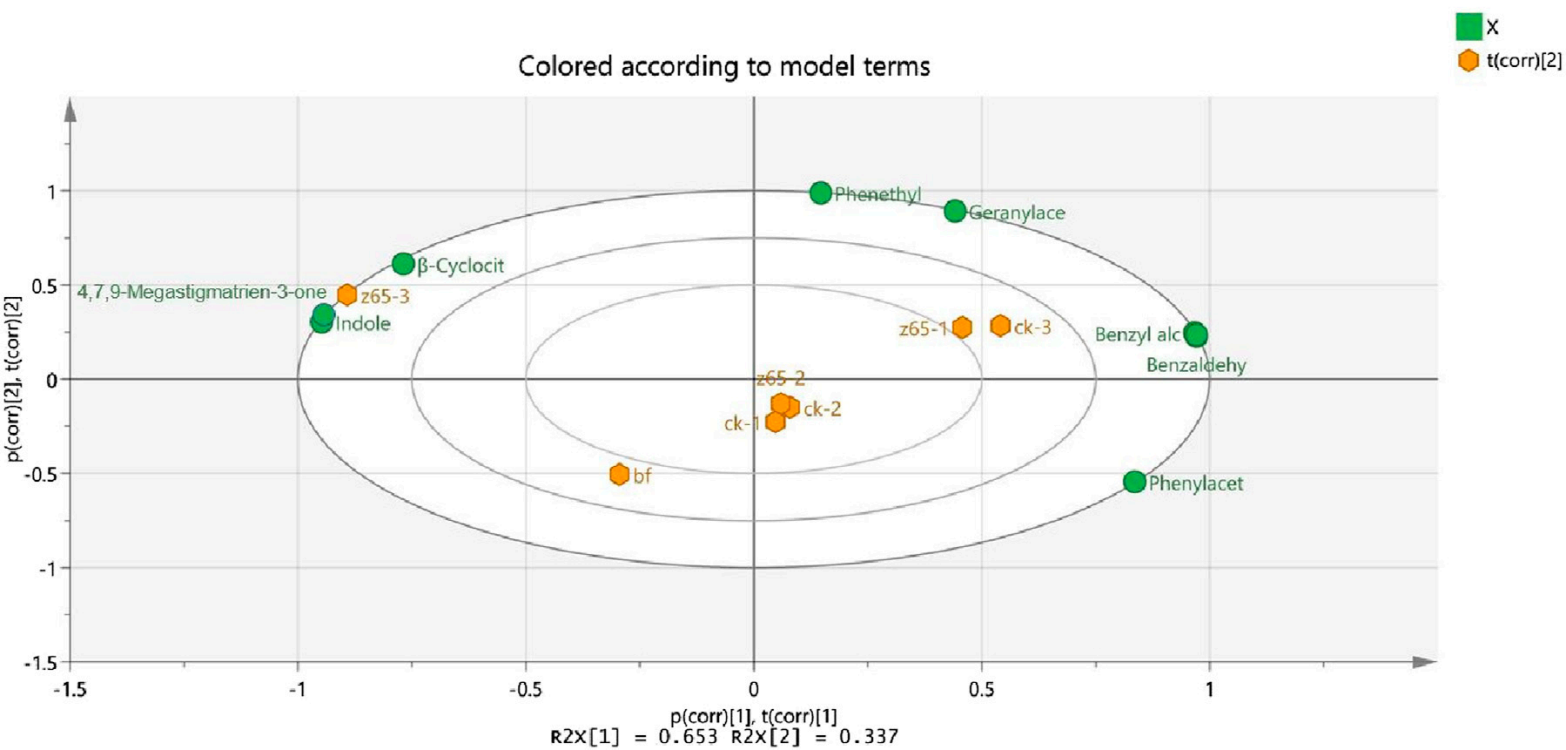
Principal component analysis of aroma compounds with OAV >1.

For the principal component analysis (PCA) plot of aroma compounds with odor active value >1, indole, β-ionone, and 4,7,9-Megastigmatrien-3-one exhibited higher OAV values and were identified as the main characteristic flavor compounds (Table 3), significantly influencing the sensory quality of CWLs in z65 group (Figure 1). Among these, β-ionone and 4,7,9-Megastigmatrien-3-one are carotenoid degradation products that contribute fruity and fresh aromas. Yang et al. (2024) elucidated the differences in aroma composition and style characteristics among six different origins of CTLs. They conducted GC-MS and OAV combined analysis on CTLs from different origins and ultimately identified odor descriptors such as phenethyl alcohol, consistent with the prominent aroma characteristics of cigars.

**TABLE 3:** Odor Activity Value (OAV) for volatile aroma compounds.

Aroma compounds	Threshold	OAV (mg/kg)						
		bf	Ck-1	Ck-2	Ck-3	z65-1	z65-2	z65-3
Benzaldehyde	0.75	3.40	3.97	4.49	8.23	3.25	2.33	1.33
Phenylacetaldehyde	0.63 × 10^−2^	2,255.56	7,861.90	10,260.32	6,180.95	7,668.25	8,096.83	ND
Phenethyl alcohol	0.48	12.07	13.95	40.77	33.95	72.48	95.51	101.90
β-Cyclocitral	0.03 × 10^−1^	133.33	190.00	610.00	73.33	313.33	ND	590.00
Indole	0.01	717.27	1,544.55	1,390.00	ND	ND	1790.91	1,550.00
Geranylacetone	0.06	23.50	28.83	56.17	ND	49.67	114.50	76.00
Benzyl alcohol	2.55	ND	ND	1.24	1.67	ND	ND	1.99
4,7,9-Megastigmatrien-3-one	3.86 × 10^−3^	4,914.51	18,590.67	22,761.66	15,290.16	18,735.75	25,095.85	42,059.59

### 3.4 Effects of different treatments on sensory quality of CWLs

The sensory evaluation indicators of fermented CWLs, except for burning properties and grayness, have been improved to varying degrees after different processing methods (Table 4). The smoking quality of CWLs is significantly improved after the addition of FHJ compared with the natural fermentation group. As the fermentation time increases, the strength of CWLs decreases, while the aroma quality and aroma quantity scores increase. When the fermentation process of CWLs enters the later stage, the changes in various scoring indicators are not significant. At this time, the main factors affecting the smoking quality of CWLs are the fermentation medium. z65-3 obtained the highest score and the greatest sensory improvement in all treatment methods. This may be due to the change in the types of aromatic substances in the CWLs after the addition of FHJ, which has a mitigating effect on the stimulation of the CWLs.

**TABLE 4:** The sensory quality of tobacco leaves after different treatments.

Treatment		Aroma quality	Aroma quantity	Concentration	Impurities	Strength	Stimulation	Aftertaste	Burning	Grey	Total
		9–0	9–0	9–0	9–0	9–0	9–0	9–0	9–0	9–0	9–0
Bf		5.8	6.5	6.1	5.7	7.5	5.0	6.2	8.0	8.0	6.0
Ck	ck-1	6.0	7.0	6.2	5.9	7.0	4.8	6.2	8.0	8.0	6.2
	ck-2	6.3	7.2	6.5	6.2	7.2	5.2	6.5	8.0	8.0	6.5
	ck-3	6.8	7.5	6.8	6.5	7.2	5.3	6.5	8.0	8.0	7.0
z65	z65-1	6.4	7.3	6.6	6.3	6.8	5.4	6.5	8.0	8.0	7.5
	z65-2	6.8	7.8	6.9	6.5	7.0	5.7	6.7	8.0	8.0	7.7
	z65-3	7.2	8.0	7.0	6.9	7.0	5.9	6.9	8.0	8.0	8.0

### 3.5 Changes in microbial community structure and their impact on the quality of CWLs

#### 3.5.1 α-Diversity

**FIGURE 2:**
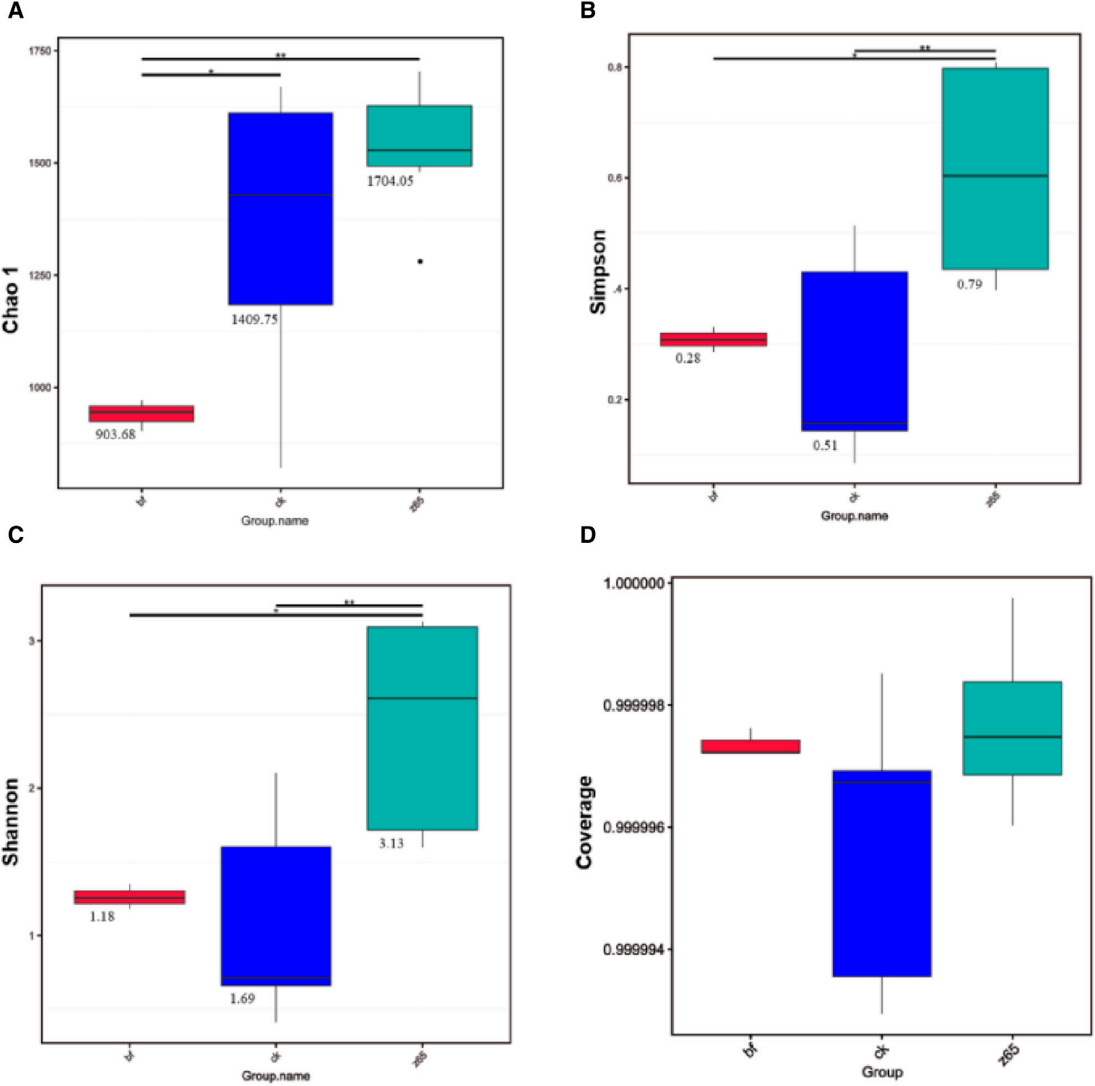
α Indices **(A)** Chao1 index, **(B)** Shannon index, **(C)** Simpson index, **(D)** Coverage).

Alpha diversity analysis was conducted on the microbial communities present on the surface of CWLs. The microbial coverage of all CWLs samples was close to 1 (Figure 2D), indicating high coverage and reasonable sampling, suggesting that the sequencing results could accurately reflect the distribution of microbial communities on the surface of CWLs. The Chao1 index reflects the richness of microbial communities, while the Shannon index and Simpson index reflect the diversity of microbial communities. The Chao1 index showed that the bacterial community richness in the z65 group was higher than that in the bf group and significantly higher than that in the ck group (Figure 2A). Additionally, the bacterial community diversity in the z65 group was higher than that in the ck group and significantly higher than that in the bf group (Figures 2B, C). Thus, the addition of FHJ effectively enhances the richness and diversity of bacterial communities on the surface of CWLs.

#### 3.5.2 Number of OTUs

**FIGURE 3:**
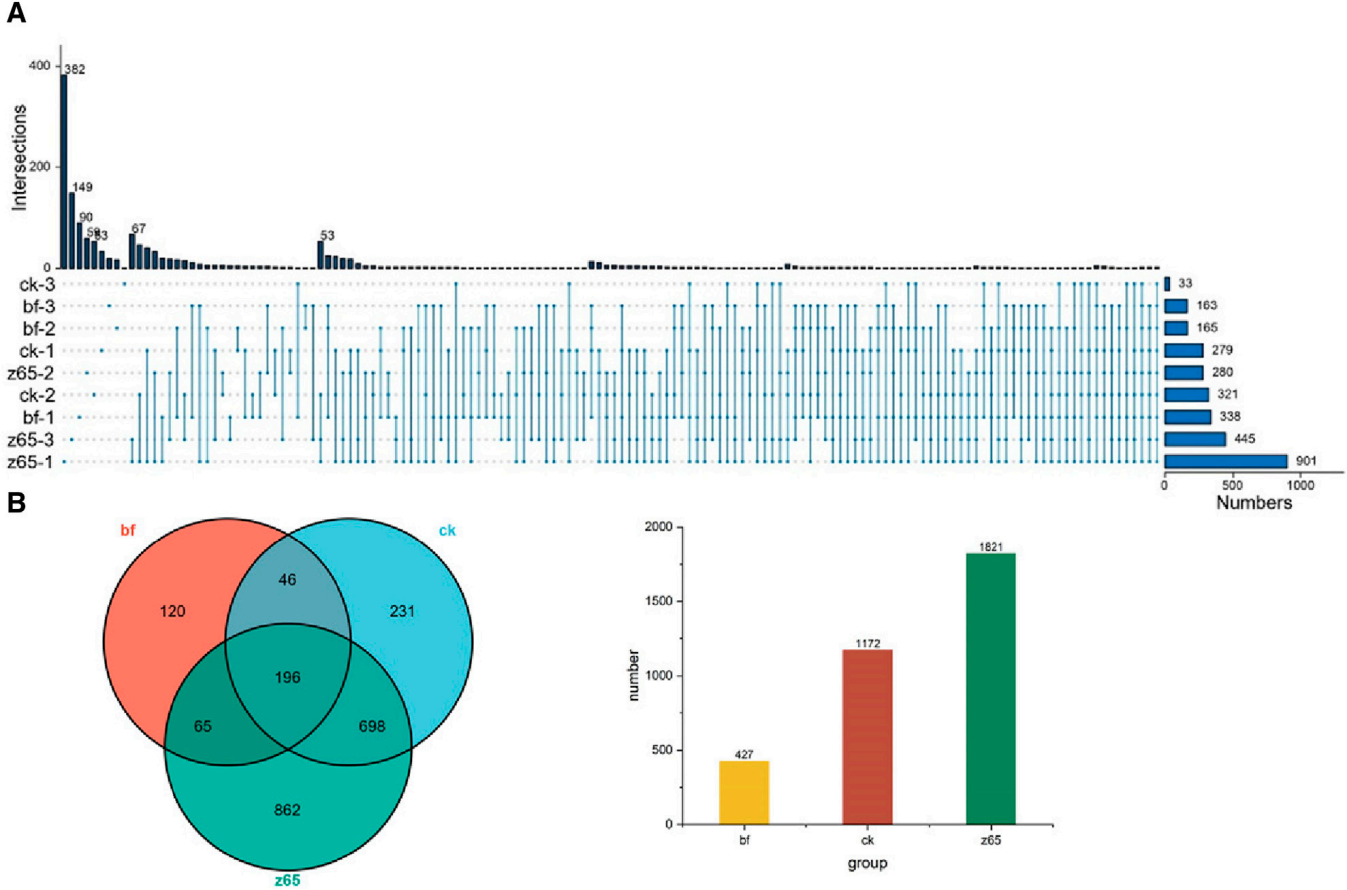
Upset plot of bacterial **(A)** and OTU levels in cigar tobacco leaves **(B)**.

Upset plots and Venn diagrams were constructed at the OTU level based on the surface microorganisms of CWLs to describe the distribution of microbial community composition in the present study, as shown in Figure 3. It was showed that the number of OTUs on CWLs ranged from 427 to 1,821. The number of unique OTUs in the z65 group was significantly increased to 862 compared to the bf (120) and the ck (231) groups. The Upset plot depicted the number of OTUs of surface microorganisms on CWLs in different turning groups. The results indicated that the z65-3 contained 445 OTUs, with 149 unique OTUs. It can be inferred that fermentation treatment can effectively increase the number of microorganisms on the surface of CWLs compared to the bf group. Moreover, the number of bacterial OTUs in the z65 group was significantly increased compared to the ck group, suggesting that the dynamic changes in the microbial community structure on CWLs are related to exogenous additives. After changing fermentation conditions, the number of OTUs on CWLs ' surfaces increased, indicating that addition of exogenous substances can enhance the number of OTUs on CWLs.

#### 3.5.3 Changes in bacterial community structure of CWLs

**FIGURE 4:**
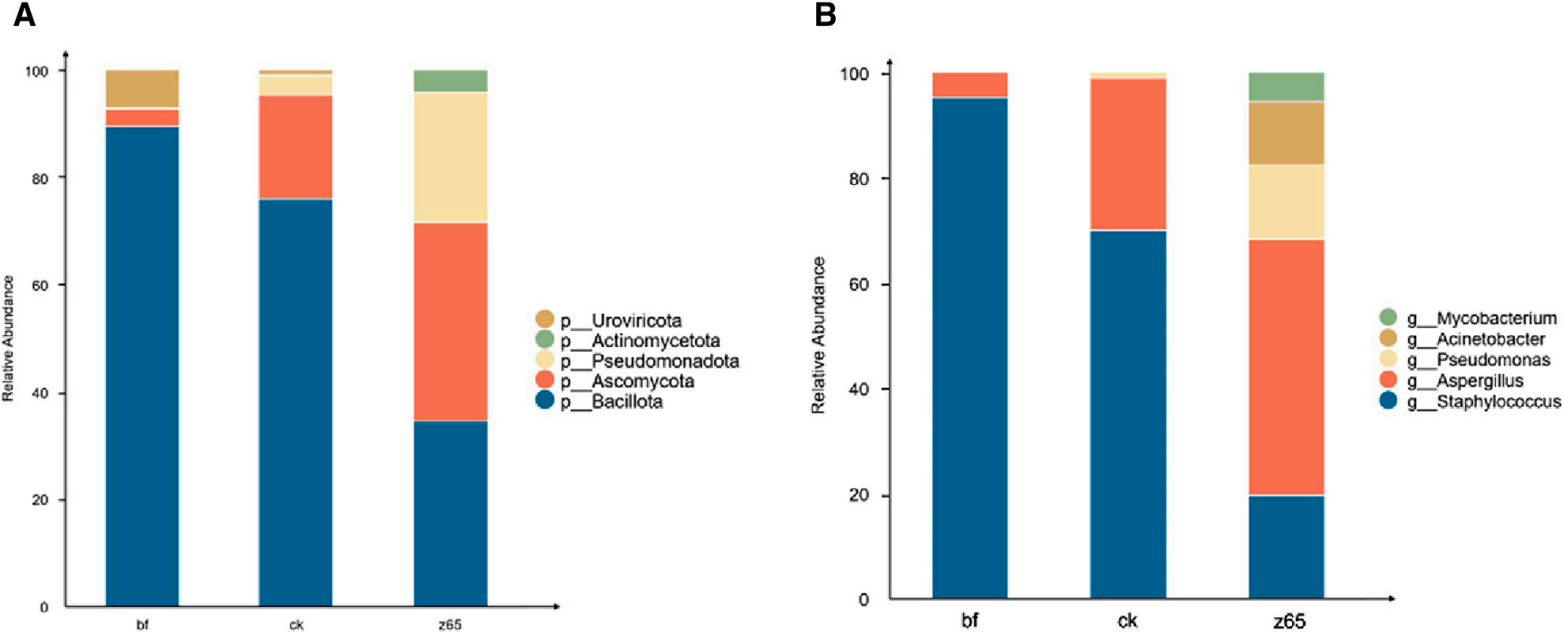
Microbial community composition of CWLs at phylum **(A)** and genus **(B)** levels.

The growth and metabolism of microbial communities play a significant role in the quality of CWLs. At the phylum level, microbial taxa with a relative abundance exceeding 0.5% are defined as dominant phyla, while at the genus level, those with a relative abundance exceeding 1% are defined as dominant genera. As shown in Figure 4A, the microbial composition of CWLs mainly consists of four phyla: Bacillota, Ascomycota, Pseudomonadota, and Actinomycetota, with Bacillota and Ascomycota being the dominant phyla. In the bf group, Bacillota exhibited the highest abundance as the dominant phylum. In the ck group, the abundance of Bacillota decreased while that of Ascomycota increased. However, Bacillota still maintained absolute dominance, accounting for over 75% of the total abundance. In the z65 group, the proportion of Bacillota continued to decline, while the proportions of Ascomycota and Pseudomonadota increased. Additionally, Actinomycetota, which was distinct from other treatment groups, appeared in this group. The overall species richness increased in the z65 group, with a more balanced species distribution.

The microbial structure of CWLs mainly comprises five genera (Figure 4B): *Staphylococcus*, *Aspergillus*, *Pseudomonas*, *Acinetobacter*, and *Mycobacterium*. In the bf group, *Staphylococcus* predominated, accounting for over 90% of the total abundance. In the ck group, after fermentation, the proportion of *Staphylococcus* decreased while that of *Aspergillus* increased. In the z65 group, there was a significant decrease in the proportion of *Staphylococcus*, with *Aspergillus* replacing it as the dominant genus. Additionally, the proportion of *Pseudomonas* increased, and unique genera such as *Acinetobacter* and *Mycobacterium* appeared in the z65 group.

**FIGURE 5:**
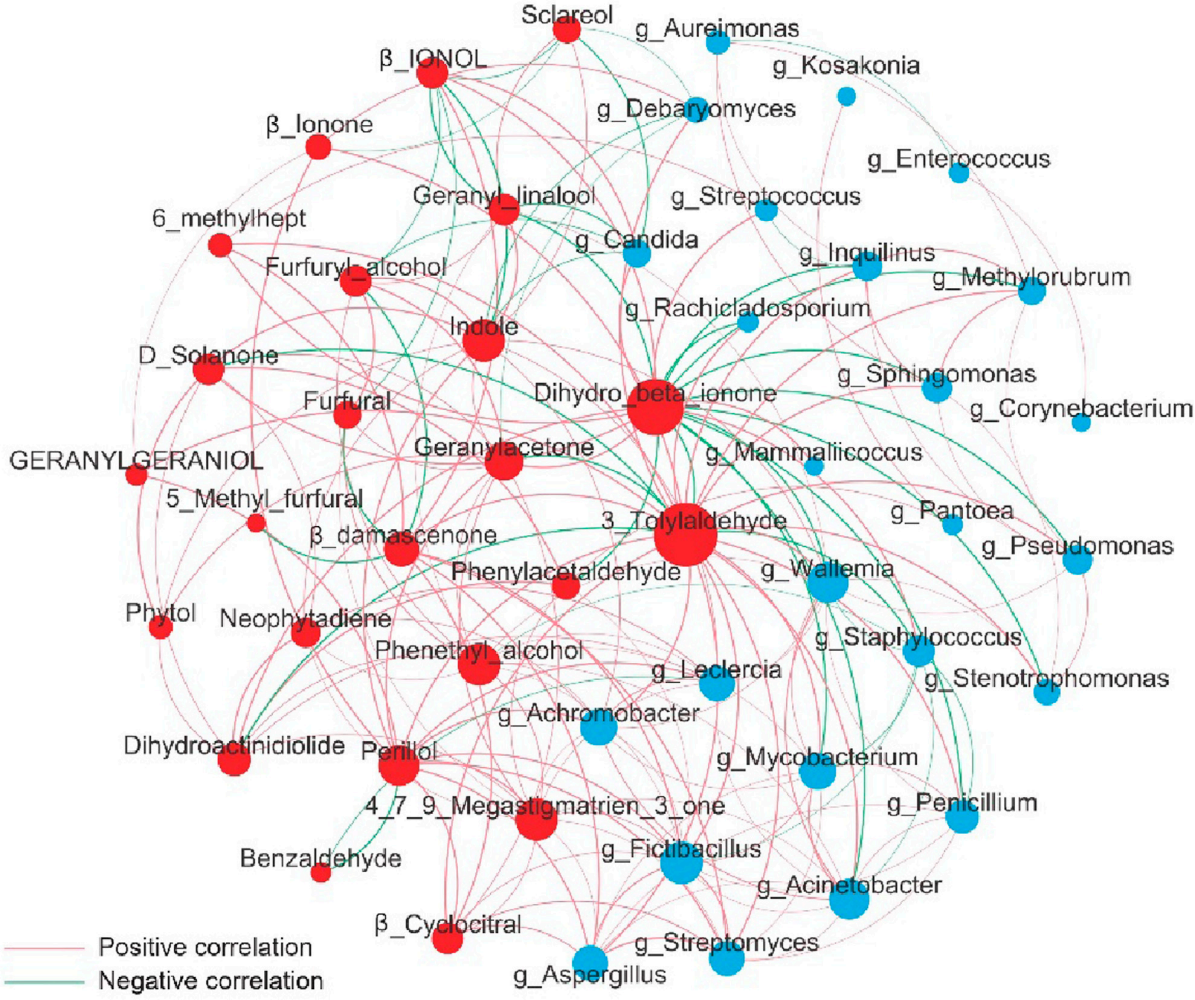
Correlation network analysis among different microbial at genera levels.

The interaction between microorganisms at different taxonomic levels is depicted in Figure 5. The size of the dots represents the species abundance, while the color of the dots represents the phylum to which the species belongs. The lines connecting the dots indicate the interaction between species, with red indicating synergy and blue indicating antagonism. In the z65 group, there was an increase in the abundance of the phylum Ascomycota and a decrease in the abundance of the phylum Bacillota. This shift may be attributed to an antagonistic relationship between Bacillota and Ascomycota.

#### 3.5.4 Predicted metabolic functions of microbiological communities in CWLs

**FIGURE 6:**
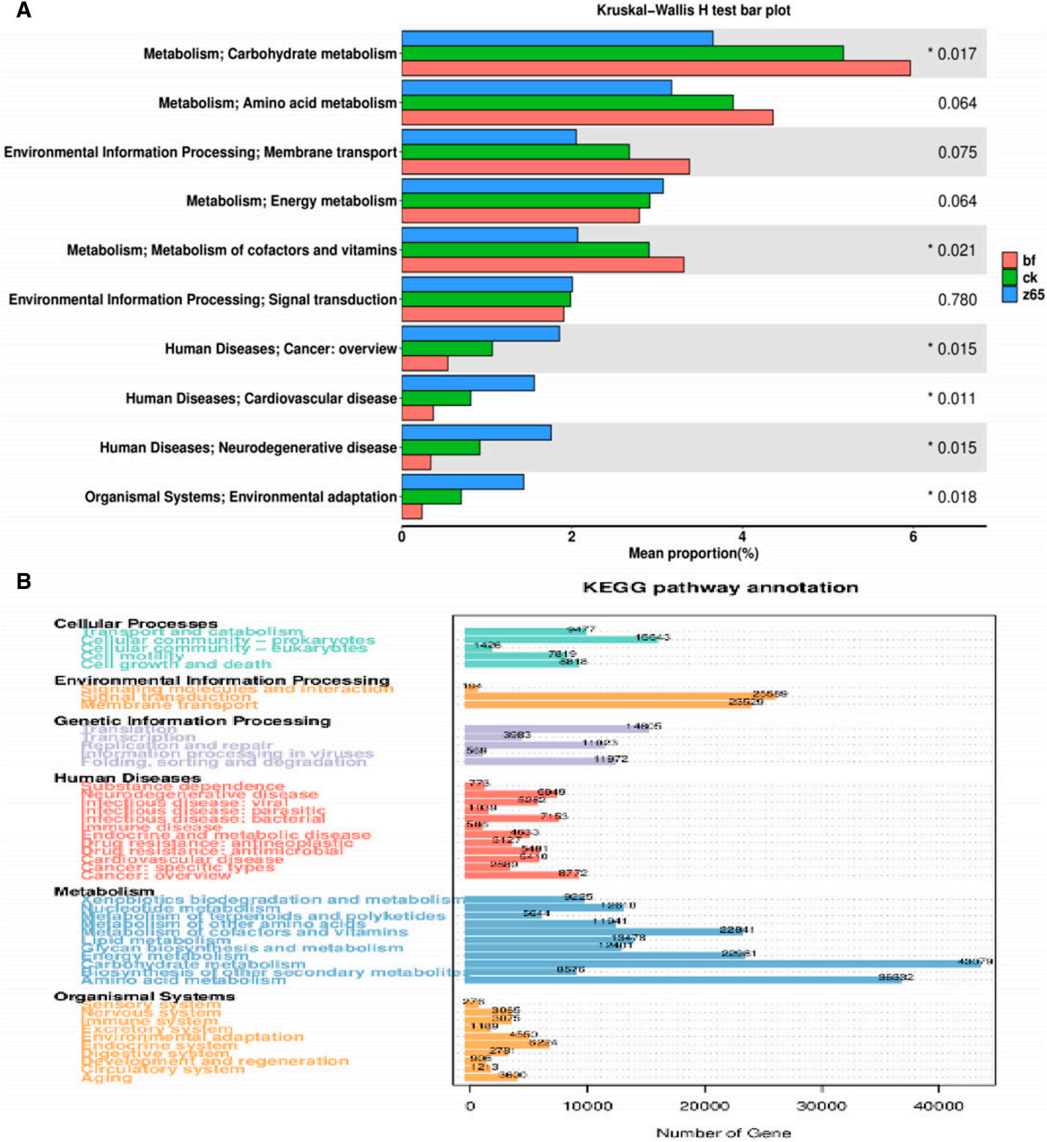
Analysis of species contributions to KEGG **(A)** and the number of genes related to KEGG metabolic pathways **(B)**.

KEGG is a database for analyzing biological metabolic pathways, primarily integrating gene and genome information with higher-level functional information to systematically analyze gene function (Aoki-Kinoshita and Kanehisa, 2007). The microbial community structure of CWLs undergoes significant changes in different treatment groups, leading to alterations in community metabolic pathways. The species contribution to KEGG analysis is shown in Figure 6A. The top five pathways in relative abundance of microbial metabolism functions in CWLs communities include carbohydrate metabolism, amino acid metabolism, membrane transport, energy metabolism, and metabolism of cofactors and vitamins. Differences in the abundance and diversity of bacterial communities in CWLs for different purposes lead to differences in the formation of functional genes, thereby affecting metabolic pathways and ultimately influencing the taste of CWLs. Intermediate products of carbohydrate metabolism pathways can provide raw materials for the synthesis of many substances, such as the synthesis of aromatic amino acids.

#### 3.5.5 Differential microorganisms in CWLs under different treatment methods

**FIGURE 7:**
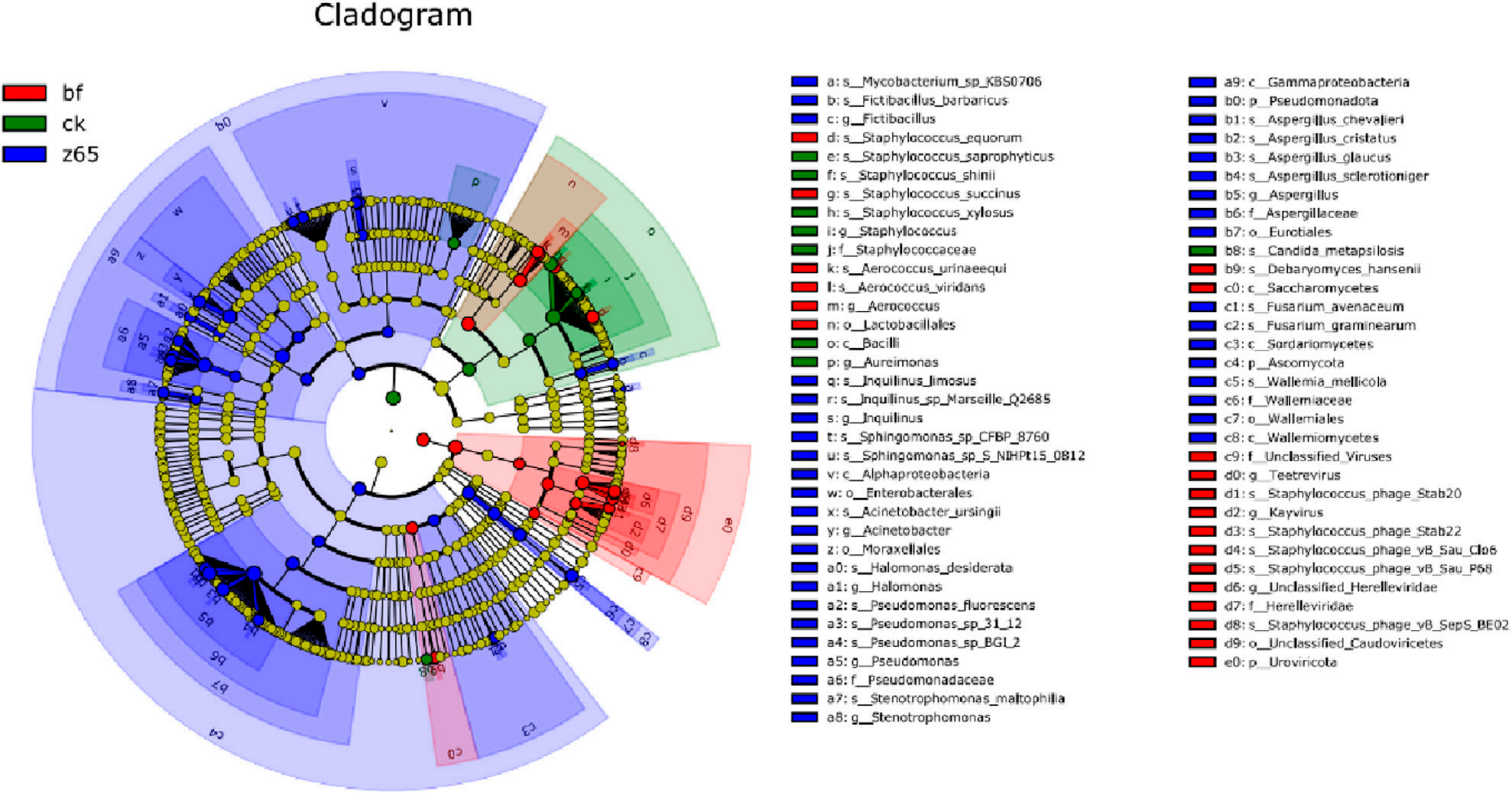
Differential microbes in CWLs under different treatment conditions.

LEfSe analysis revealed significant differences in microbial composition among different treatment groups of CWLs, as shown in Figure 7. The evolutionary branch diagram displays microbial classification from phylum to species, with different colors representing significantly different microbes in CWLs under different treatment conditions. Microbes with significant differences among CWLs in different treatment groups are distributed across three phyla, six classes, six orders, six families, 13 genera, and 33 species. Specifically, in the bf group, significantly different phyla includes Uroviricota, and different genera includes *Aerococcus*, *Teetrevirus*, and *Kayvirus*. *Staphylococcus* and *Bacillus* are positively correlated with the content of perilla aldehyde and isovalerone, respectively (Mostafa et al., 2022). In the z65 group, more significantly different microbes compared to other treatment groups indicated a greater difference from other treatment groups. These results suggest that the addition of FHJ can increase significantly different microbes in CWLs, playing an important role in improving the quality of CWLs.

#### 3.5.6 Correlation analysis between microbial community and conventional chemical components, aromatic compounds in CWLs

**FIGURE 8:**
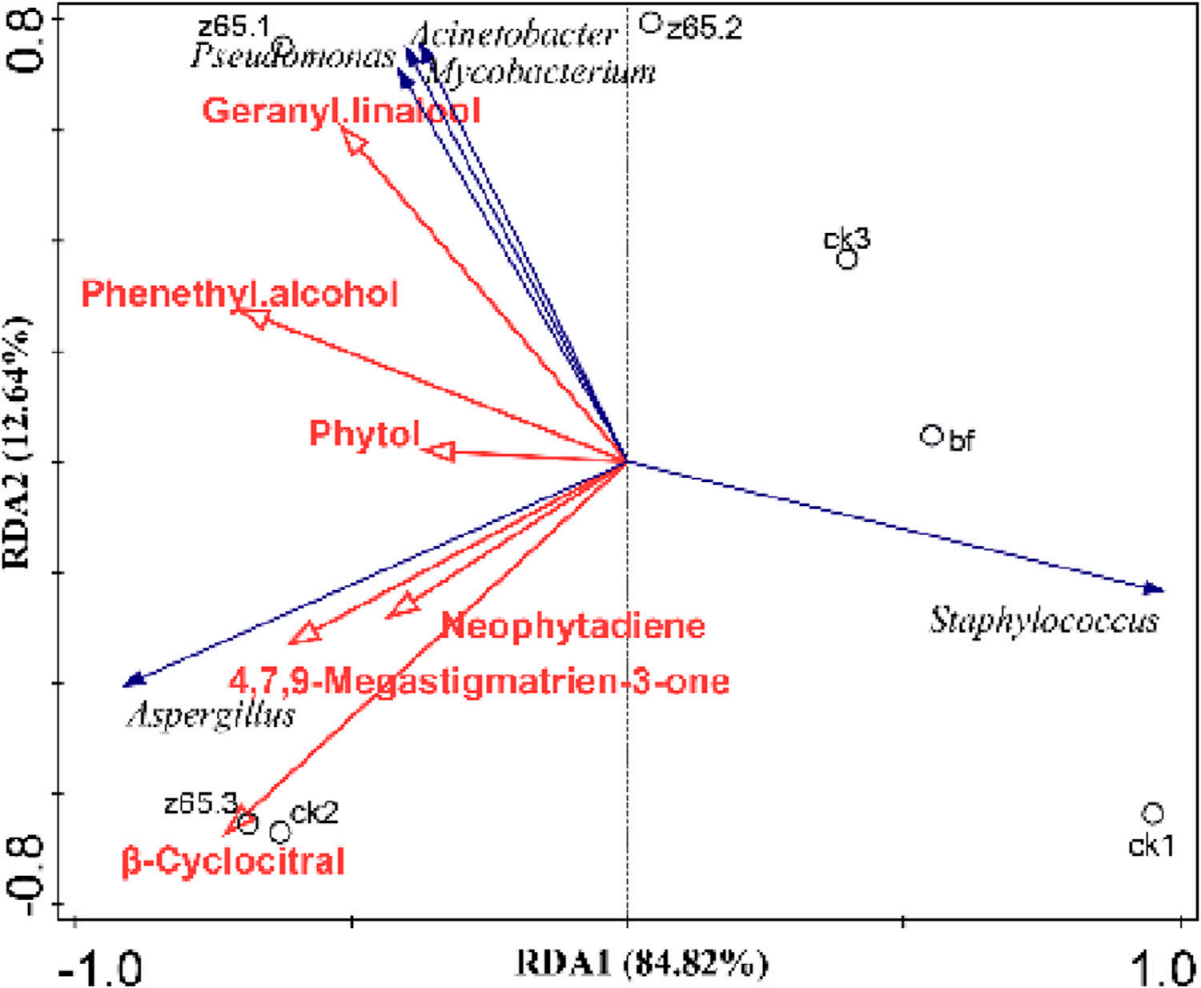
Redundancy Analysis (RDA) of dominant bacteria on CWLs surfaces and aroma compounds.

To explore the relationship between the microbial community structure on the surface of CWLs, aroma compounds and different treatment groups, redundancy analysis (RDA) was conducted. Dominant microbes and aroma compounds were used as response variables, while experimental sampling samples were used as explanatory variables. As shown in Figure 8, the explanatory variances of the samples for canonical axes one and two were 84.82% and 12.64%, respectively. The second and third quadrants were better explained by the selected environmental factors. Among them, the vector weights of aroma compounds 4,7,9-megatetraen-3-one and β-citral for z65 group were relatively high, consistent with the results of the OAV analysis. Moreover, the levels of these two substances were closely related to *Aspergillus*. *Aspergillus*, *Pseudomonas*, and *Acinetobacter* were synergistically associated with significant aroma compounds and exhibited antagonistic interactions with *Staphylococcus*, resulting in a significant decrease in the proportion of *Staphylococcus* in the z65 group (Figure 5).

The generation of 4,7,9-megatetraen-3-one and β-citral is associated with *Aspergillus* and has the greatest impact on the results of the third turning of the z65 group. The production of linalool, an aromatic compound, is related to *Acinetobacter*, *Pseudomonas*, and *Mycobacterium*, and has the greatest impact on the results of the first turning of the z65 group. This is consistent with the changes in the content of volatile aroma compounds shown in Table 2.

## 4 Discussion

The fermentation of CX-026 CWLs from Enshi, Hubei Province, China, after adding FHJ, produced in the Qinba region of China, was studied in the present study. After 40 days of fermentation, the changes in aroma compounds and microbial community structure of CWLs, as well as their correlation, were analyzed. Additionally, alterations in relevant microbial metabolic pathways were investigated. The results indicate that the addition of FHJ significantly influences the flavor quality and microbial community structure of CWLs during the fermentation process. FHJ, as an exogenous additive, co-ferments with CWLs, plays a crucial role in enhancing the sensory quality of the cigar tobacco leaves. During rice wine fermentation, the Maillard reaction produces compounds such as furfural and benzyl alcohol (Yu et al., 2022). The content of furfural is positively correlated with the content of reducing sugars, playing a promoting role in improving the taste of Huangjiu (Gao et al., 2024). The incorporation of FHJ introduces its inherent aroma compounds into the fermentation process. Moreover, the acidic pH of FHJ alters the fermentation environment of the CWLs, promoting the pathway of carotenoid degradation and thereby increasing the content of volatile aroma compounds in the CWLs (Li et al., 2023). Additionally, the addition of FHJ induces changes in the original microbial community structure of CWLs, resulting in variations in both the types and contents of volatile aroma compounds.

A total of 34 volatile aroma compounds were detected via GC-MS analysis. The content and types of volatile aroma compounds in CWLs fermented through different treatment methods were different, with the formation of aroma characteristics depending on various factors such as fermentation conditions and the addition of exogenous microorganisms (Yao et al., 2022b). In the z65 group, the levels of 4,7,9-Megastigmatrien-3-one, Geranylacetone, Benzaldehyde, D-Solanone, Neophytadiene, Sclareol, Phytol, and P-vinylguaiacol were significantly elevated compared to those in the bf group. Moreover, the levels of Furfural, Geranyl linalool, and P-vinylguaiacol were significantly increased compared to those in the ck group. Dihydroactinidiolide and Phenethyl alcohol levels were notably increased compared to both the bf and ck groups. Alcoholic substances are byproducts of yeast metabolism, with the highest concentration among all categorized aroma substances. The generation mechanism of alcoholic substances primarily involves two pathways: glucose synthesis and the decomposition metabolism of corresponding amino acids. FHJ contains various amino acids and plays a promoting role in the generation of corresponding alcoholic substances (Gao et al., 2024). Thus, phenethyl alcohol, a degradation product of aromatic amino acids known for its sweetness and contribution to rose-like aroma (Liu et al., 2022), increased from 5.78 μg/g to 48.81 μg/g after FHJ fermentation. FHJ itself contains phenethyl alcohol, which was possibly introduced into CWLs through the addition of FHJ. Alternatively, the addition of FHJ may have altered the microbial community of CWLs, leading to an increase in the production of phenethyl alcohol. Furthermore, significant changes were observed in the levels of carotenoid degradation products such as 2,5-Dihydroxy-3-undecylcyclopentanone and 4,7,9-Megastigmatrien-3-one, which exhibited a significant positive correlation with aroma quality and aftertaste indicators, and had a significant favorable effect on irritant indicators (Wang et al., 2024). In the z65 group, the content of 4,7,9-Megastigmatrien-3-one significantly increased compared to bf and the ck groups (from 18.97 μg/g to 162.35 μg/g). 4,7,9-Megastigmatrien-3-one, a carotenoid degradation product, exhibits a licorice-like sweet aroma, enhancing the fullness, sweetness, and tobacco aroma of the smoke, improving the off-flavor, and rendering the smoke smoother (Zhang et al., 2024). Similarly, the content of neophytadiene increased from 934.32 μg/g to 1,883.70 μg/g. Neophytadiene is a degradation product of chlorophyll and is the component with the highest content among volatile substances in CWLs. It can soften and smoothen the smoke while reduce its irritability, and also has a promoting effect on other volatile aroma components in CWLs entering the smoke (Hu et al., 2022c). Indole, a Maillard degradation product associated with tryptophan metabolism, contributes a floral aroma to CTLs, enhancing the smoothness of the smoke (Li et al., 2023). Although other aroma substances are classified as other reaction degradation products, they also have a promoting effect on enhancing the aroma and quality of CWLs. The content of carotenoid degradation products and Maillard reaction products increased significantly compared to bf and ck groups. Carotenoids are enzymatically converted into non-volatile aroma substance precursors, which are then released as volatile aroma substances. FHJ provides an acidic environment for CWLs fermentation, promoting the degradation pathway of carotenoids (Li et al., 2023). In the study by Yao et al. (2022a), the addition of exogenous strain H1 to CTLs fermentation resulted in a doubling of carotenoid degradation product content compared to the control group. Carotenoid degradation products are mainly ketones. Due to microbial community structure changes of CWLs after FHJ addition, carotenoid degradation reactions was enhanced, thus promoting the quality of CWLs (Wang et al., 2024). Degradation products of aromatic amino acids, such as benzaldehyde and phenethyl alcohol, can effectively contribute to floral and sweet aromas, making the smoke mild (Ma et al., 2023). Genes associated with amino acid metabolism pathways dominate in quantity (Figure 6B). Amino acid metabolism can undergo Maillard reactions with reducing sugars, which are closely related to the sensory quality of cigar (Zhao et al., 2023). FHJ contains a variety of amino acids in abundance, providing precursor substances for the Maillard reaction. Zong et al. (2023) added cocoa substrate to CTLs fermentation, allowing them to co-ferment. Sensory evaluation and analysis of aroma substance quantity showed that the addition of cocoa substrate made the legume aroma of CTLs more prominent, increased the sweetness of CTLs, and also increased the content of aromatic amino acids, as well as significantly enhanced the total quantity of aroma substances and carotenoid degradation product content.

In the ck group, there were no significant differences at the phylum level, while significantly different genera include *Staphylococcus* and *Aureimonas*. The z65 group exhibits a greater number of unique OTUs, and significant changes occur in the richness and diversity of the microbial community, significantly different phyla includes *Fictibaillus*, *Inquilinus*, and *Aspergillus*. Compared to the ck group, the addition of FHJ facilitates the process of microbial community structure succession, leading to a significant decrease in the proportion of *Staphylococcus* and an increase in the proportions of *Aspergillus*, *Pseudomonas*, and *Acinetobacter*. Wu et al. (2023) analyzed the microbial community structure and volatile aroma components during the pile fermentation of CTLs, showing that different community structures affect microbial metabolism, and different microbes are significantly correlated with volatile aroma compounds. *Aspergillus*, during FHJ fermentation, actively contributes to the flavor formation and enhancement of CWLs. The reduction in the irritancy of CWLs may be attributed to the role of *Aspergillus* in degrading proteins, starches, and other large molecular substances into peptides and other smaller molecules, *Aspergillus* is a dominant fungus in the degradation of terpenoids and higher fatty acids, while *Pseudomonas* can degrade nicotine, thereby improving the sensory quality of the CWLs (de Vries, et al., 2001; Ye et al., 2017).

The fermentation process led to varying degrees of change in microbial community structure (Yao et al., 2023). These changes indicate that the addition of FHJ altered the growth environment of microbial communities on CWLs, accelerated the succession of bacterial communities and impacted both the structure and abundance of microbial populations. It was found that the microbial diversity and richness of CTLs fermented with different media were increased by adding different media for fermentation (Hu et al., 2023). During CWLs fermentation, different microbial communities participate in the transformation of tobacco compounds (Wang J. Y. et al., 2024). *Staphylococcu*s, as the dominant genus, plays a crucial role in protein degradation and amine metabolism, thereby reducing the bitterness produced after smoking (Liu et al., 2021). *Acinetobacter* can produce 4,7,9-Megastigmatrien-3-one. The increased proportion of *Acinetobacter* in the z65 group resulted in a significant increase in 4,7,9-Megastigmatrien-3-one content. Consistent with Zheng’sresearch, inoculating external *Acinetobacter* into CWLs improved the smoking sensation, increased the bean and milk aroma, and reduced irritation (Zheng et al., 2022). *Mycobacterium* can degrade sterols in cigar leaves, thereby reducing the precursors of harmful substances (Liu et al., 2020). During FHJ fermentation, dominant microorganisms such as *Aspergillus* and *Rhizopus* are found. *Aspergillus* metabolizes to produce proteases and amylases, capable of hydrolyzing macromolecules such as proteins and carbohydrates into peptides and amino acids, thereby generating a large number of flavor compounds and reducing the irritant taste of cigars (Xue et al., 2023). Metabolic pathways were closely related to cigar flavor formation include carbohydrate metabolism and amino acid metabolism (Geng et al., 2023). A secondary pathway abundance analysis of CWLs, fillers, and binders from different producing regions was conducted before, it was showed that the samples were mainly enriched in carbohydrate metabolism, amino acid metabolism, and energy metabolism (Fan et al., 2023). The acidic pH of FHJ creates an acidic environment conducive to the growth of *Aspergillus*. Thus, the addition of FHJ promotes the enrichment of *Aspergillus* in CWLs, contributes to the improvement of CWLs quality. Certain microorganisms, such as *Bacillus*, can participate in metabolic activities such as nitrate respiration, nitrate reduction, and nitrogen respiration (Chen et al., 2010), eliminating the inherent pungent odor of CWLs and promoting the formation of aroma.

The sensory changes in CWLs are not only related to the addition of FHJ but also influenced by the interaction between endogenous microorganisms in CWLs and exogenous additives. The addition of exogenous substances alters the original microbial community structure of CWLs, leading to changes in conventional chemical composition and volatile aroma compound content and types, thereby affecting the smoking quality of CWLs. Additionally, the addition of FHJ can effectively enhance the richness of bacteria on CWLs. This finding is consistent with the results of Zhang et al. (2023), indicating that changes in fermentation conditions, including temperature, humidity, and exogenous additives, have different effects on the microbial community structure and species richness of CWLs. In this study, the microbial community structure of the naturally fermented group did not show significant changes compared to the bf group, while the microbial community structure of the z65 group differed significantly from both the unfermented and naturally fermented groups. It is inferred that the addition of FHJ significantly affects the quality of CWLs and can effectively enhance their quality.

## 5 Conclusion

When traditional fermentation products fail to meet demands of consumer, the addition of exogenous substances can effectively enhance product quality. Co-fermentation of FHJ with CWLs significantly promotes the CWLs sensory quality and the microbial community structure on the surface of CWLs. The incorporation of FHJ introduces its own volatile aroma compounds, leading to a significant increase in the formation of aroma compounds in CWLs. Furthermore, it alters the original fermentation environment of CWLs, resulting in changes in the microbial community structure. The dominant bacterial genus transitions from a single *Staphylococcus* genus to *Staphylococcus*, *Aspergillus*, *Pseudomonas*, and *Acinetobacter*. Through fermentation, the z65 group exhibits a substantial increase in significantly different microbial species, thereby alters the types and levels of volatile aroma compounds in CWLs and consequently significantly improves the sensory quality of CWLs. The addition of FHJ can effectively improve the quality of CWLs and reduce their harshness, making the smoke smoother and more delicate when burned. This co-fermentation process not only preserves the original rich taste and complex layers of CWLs, but also cleverly blends the mellow aroma of FHJ. CWLs enthusiasts will experience an unprecedented taste enjoyment when savoring CWLs infused with FHJ, as the smoothness of the smoke intertwines with the mellowness of the wine aroma.

## Data Availability

The raw data supporting the conclusions of this article will be made available by the authors, without undue reservation upon request. Sequence data associated with this project have been deposited in the NCBI Short Read Archive database (Accession Number: PRJNA111063).
